# Innovation in Psychotherapy, Challenges, and Opportunities: An Opinion Paper

**DOI:** 10.3389/fpsyg.2019.00495

**Published:** 2019-03-19

**Authors:** Janina Isabel Schweiger, Kai G. Kahl, Jan Philipp Klein, Valerija Sipos, Ulrich Schweiger

**Affiliations:** ^1^ Department of Psychiatry and Psychotherapy, Medical Faculty Mannheim, Central Institute of Mental Health, University of Heidelberg, Mannheim, Germany; ^2^ Klinik für Psychiatrie, Sozialpsychiatrie und Psychotherapie, Medizinische Hochschule Hannover, Hannover, Germany; ^3^ Klinik für Psychiatrie und Psychotherapie, Universität zu Lübeck, Lübeck, Germany

**Keywords:** psychotherapy development, psychotherapy innovation, randomized controlled clinical trails, metacognitive therapy (MCT), scientific base of psychotherapy

## Abstract

Psychotherapy as a field tends toward conservativism, and the rate of innovation and development of new evidence-based effective treatments has been slow. The paper explores important barriers to innovation like the dodo bird verdict and the habit of starting the development of therapeutic methods from techniques. The paper looks at the opportunities for translating basic science in psychology into psychotherapeutic techniques. Metacognitive therapy stands out from other psychotherapies by its development from basic science. The paper describes the development of the techniques detached mindfulness and attention training, how they were derived from basic science and tested for their suitability in the therapy of patients with anxiety disorders. By this process, metacognitive therapy may be an important model for the innovation process in psychotherapy.

## Introduction

The implementation of psychotherapy in general healthcare has been one of the significant innovations of the twentieth century and has revolutionized how the health care system deals with mental disorders. Psychotherapy is an essential focus of training in clinical psychology and physicians aiming for board certification in psychiatry or psychosomatics in many countries. Despite this transformative impact, the rate of innovation and development of new evidence-based effective treatments has been slow, and it has been noted that compared with medication psychotherapy use is on the decline in the US (Gaudiano and Miller, 2013). This opinion paper examines some of the barriers to innovation that we believe have slowed progress. It discusses alternative ways of fostering innovation and uses the development of metacognitive therapy by Wells and colleagues as an example of a strategy that overcomes barriers and discusses how MCT fits into current assumptions about innovation.

## Barriers

### The Therapeutic Relationship and the Dodo Bird Verdict

One of the widespread assumptions in psychotherapy is that a good therapeutic relationship is the critical mechanism of successful psychotherapeutic treatment (Wampold, 2015). It is assumed that the relationship is more significant than the underlying model of causality and the manipulation of its causal variables and is the universal change mechanism uniting all psychotherapy approaches. This way of thinking postulates that creating expectations through explanations of the disorder and the treatment involved and the enactment of health-promoting actions are further common factors. The presumed equivalence of all therapies after correction for the therapeutic relationship has resulted in the dodo bird verdict (Luborsky et al., 2002). Based on the finding in meta-analyses that a broad spectrum of psychotherapeutic treatments in depression is similarly effective, Cuijpers has claimed that there is a possibility to minimize the number of existing therapies (Cuijpers, 1998). However, results of meta-analyses support differences between psychotherapies (Budd and Hughes, 2009; Tolin, 2010).

While the patient-rated quality of the therapeutic alliance is a good predictor of outcome in therapy (Cameron et al., 2018), a meta-analysis of the relationship between therapeutic alliance and treatment outcome in eating disorders showed that the association between alliance and outcome is weaker than the association between early symptom improvement and later alliance (Graves et al., 2017). Thus, it would seem that early symptom improvement affects the later alliance. We might presume that the most effective treatments give rise to the strongest alliances. What is lacking are experimental studies that actively manipulate therapeutic alliance, and so the evidence remains restricted to longitudinal predictor analyses that can do little more than implying causal relations (Fluckiger et al., 2018). Despite the lack of experimental evidence, the prevalent assumption is that a good working alliance is “a thing” that resides in the interpersonal harmony between two persons, providing a patient with a healing experience that appears to be part of a stable, benign relationship. Related to this idea is the presupposition that some therapists “have it” while others do not, meaning that there are good and bad therapists, as categories. Unfortunately, this explanation falls short of the alternative but little-tested assumption that a good therapeutic relationship is an emergent phenomenon produced by professionalism, plausible models, and experience of change already early in therapy.

Consistent with the assumption that the alliance is, in fact, an emergent factor of effective therapy, the working alliance in pure Internet therapy is remarkably good (Heim et al., 2018). The continued perception of the therapeutic relationship as the primary underlying factor of psychotherapy effectiveness is a barrier because it reduces the necessity of developing innovative theories and techniques since new techniques only make a marginal difference. Assigning the therapeutic relationship to the role of the critical cause of change, instead of modeling it as an emergent phenomenon of change creates inertia in research on psychopathological mechanisms and complacency in therapists.

### Starting the Development of Therapeutic Methods From Techniques

New approaches have most often been devised based on techniques, that is on the basis of assembling combinations of treatment techniques that appear to work. Such approaches are often only loosely grounded in theoretical models, and the models of treatment mechanisms may develop after the treatments themselves.

A top-down approach in the design of technology starts with an overview of the relevant system (e.g., dysfunctional beliefs) but does not specify subsystems in sufficient detail or elucidate how they impact on functioning. For instance, negative automatic thoughts and beliefs are purported to cause or maintain disorder in the cognitive model. However, as pointed out by Wells and Matthews (Wells and Matthews, 1996), this approach does not consider broader aspects of cognition that are known to be associated with the disorder such as biases in the regulation of attention and levels of control of cognition. The cognitive-behavioral model has not advanced along with recent developments in cognitive psychology and theory such that the practice of therapy is only loosely tied to an understanding of mechanisms. Beck based CBT on the description of problematic thought content and processes of cognitive distortion in patients (Beck, 1963, 1964). The primary intervention derived from this observational approach and comprised of correcting cognitive distortions and deficiencies in schema content using Socratic dialogue. This fundamental change technique of cognitive therapy (CT) is derived from philosophy and is not rooted in or supported by experimental psychology. To the contrary, research shows that trying to replace dysfunctional thought by more appropriate thinking may result in thought suppression and have adverse paradoxical effects (Longmore and Worrell, 2007; Magee et al., 2012). Subsequently, more techniques used initially in behavioral activation, assertiveness training, anxiety management or mindfulness meditation have been incorporated to form a more eclectic cognitive behavioral therapy (CBT).

A second notable example of technique-driven development is dialectical behavior therapy (DBT). It is based on the assumption that patients with borderline personality disorder have skills deficits in emotion regulation (Linehan et al., 1991; Linehan, 2014). At the core of the interventions are approximately 50 skills that are taught to patients to improve emotion regulation. Again, learning theory informed the selection of these skills, but none was derived from experimental psychology nor were they individually tested. As packages, both CBT (Beck and Dozois, 2011) and DBT (Stoffers et al., 2012) can be considered as well supported by evidence. There were a few studies involving component analysis (Jacobson et al., 1996) showing that in the case of CBT challenging thoughts on the content level, the primary and elemental technique may not be the essential ingredient. The introduction of disorder-specific treatment methods for depression, anxiety disorders, and personality disorders beginning in the 1960s was a big step forward for psychotherapy. These new methods led to a considerable extension of the field of activities of psychotherapy toward groups that are severely ill and were traditionally underserved.

While there is evidence that these treatments offer innovation and can work, it is important to question whether the technique-driven approach of combining a range of techniques is the most effective means of treatment development. In particular, multi-component and highly eclectic treatment packages may hide detrimental effects of specific components of a treatment method (Castonguay et al., 1996). In summary, these examples show that in psychotherapy, the dominant technique-driven approach (as in other fields) has advantages but also creates serious problems.

## Opportunities

### Starting From Basic Science

All methods of modern behavior therapy refer to general learning theory (behaviorism, cognitivism, constructivism, and social cognitive theory) or information processing theory. Only two refer to a specific psychological theory derived from general psychology: metacognitive therapy (MCT) (Wells, 2009) draws on and develops the concept of metacognition as described by Flavell (Flavell, 1979). It is grounded in the self-regulatory executive function (S-REF) model, a detailed information processing model of human cognitive and affective regulation (Wells and Matthews, 1996). Acceptance and commitment therapy (ACT) refers to relational frame theory (RFT) (Hayes et al., 2001). The exact nature of the interaction between RFT and the techniques proposed by ACT is an ongoing point of discussion. For specific information, see (Zettle et al., 2016).

An essential aspect of starting from basic science is to direct therapeutic techniques at psychological mechanisms or processes and not at mental disorders which are broad concepts summarizing symptom clusters. Focusing on a specific mechanism necessarily results in a reductionistic approach. For example, MCT assumes that worry, rumination, and threat monitoring are part of a cognitive attentional syndrome (CAS) which is a core psychological process and a transdiagnostic factor across most disorders. Putting worry, rumination, and attention to threat in the center implies that psychological dysfunction such as anxiety is a product of this mechanism, and there is no need to directly address the emotion anxiety if a technique can limit the CAS.

MCT seems to be quite unusual as it exclusively developed and uses techniques that can be directly related to the parent theory, and it was developed by systematically testing the assumptions derived from this theory. MCT started with case studies demonstrating the effects of manipulating attention focus, through the attention training technique (ATT) as a means of enhancing cognitive control and disrupting the CAS (Wells, 1990), and later on the effects of attention enhancements on exposure (Wells and Papageorgiou, 1998). There is now a significant database supporting the probable efficacy of ATT (Knowles et al., 2016; Fergus and Wheless, 2018) and full MCT (Normann et al., 2014). While there are a variety of techniques intended to modify attentional focus and attentional processes in behavior therapy literature, these were often focused on reducing anxiety through distraction, rather than based on a theory linking attention to psychological causal or maintenance mechanisms. An exception is presented by work in the area of attention bias modification (ABM) based on the finding that anxiety is associated with “automatic processing” of threat-related information and in principle, such bias might be retrained (MacLeod, 2015). However, these examples of ATT and ABM appear to be among the few exceptions in the field.

### Theory-Driven Construction of Psychotherapeutic Methods

In the case of MCT and of its individual techniques such as ATT, we see a paradigmatic shift with a predominant theory-driven development of therapeutic techniques. Furthermore, the theory is firmly grounded in objective psychological science of attention (Wells and Matthews, 1996). However, we need an awareness of the potential risks involved in this system of therapy development, and we require an ongoing process of refining psychotherapy from a basic science perspective. Helpful tools may be qualitative studies examining the effects of specific psychotherapeutic techniques, and single case studies that focus on testing-isolated techniques. Essential principles of psychotherapy like “doing a few things well” or “less is more” (low complexity results in better skill acquisition, focus on key information results in better decisions) may show their advantages in further enhancing the theory-driven approach to therapy development.

Starting the construction of psychotherapeutic methods from basic science is an exception rather than a rule. However, this is not related to a lack of progress in general psychology. Actually, there is a substantial amount of new knowledge in the field with obvious relevance that awaits translation into psychotherapy techniques, e.g., knowledge about decision making (Morewedge and Kahneman, 2010), human cooperation (Rand and Nowak, 2013), heuristics (Raab and Gigerenzer, 2015), or the theory of constructed emotions (Barrett, 2017). The development of MCT presents an example of a systematic approach to theory and testing that could be emulated in developing the full potential of other psychological discoveries.

## Conclusion

Our opinion paper points to the necessity of rethinking innovation processes in psychotherapy. Psychotherapy is a significant achievement in modern health care. It needs further evolution. To this end, it still needs to overcome barriers and might benefit from a more rigorous theory-driven approach that is informed by discoveries in psychological science. Metacognitive therapy is an example of this type of approach in which an interface between cognitive psychology and applied psychology has been developed and exploited with good effect.

## Author’s Note

The idea for this opinion paper arose during a lengthy discussion after a presentation by Adrian Wells at the World Congress of Psychiatry in Berlin in October 2017.

## Author Contributions

All authors listed have made a substantial, direct and intellectualcontribution to the work, and approved it for publication.

### Conflict of Interest Statement

The authors declare that the research was conducted in the absence of any commercial or financial relationships that could be construed as a potential conflict of interest.
